# Evolution of the Vagus Nerve Stimulation (VNS) Therapy System Technology for Drug-Resistant Epilepsy

**DOI:** 10.3389/fmedt.2021.696543

**Published:** 2021-08-26

**Authors:** Pegah Afra, Bola Adamolekun, Seyhmus Aydemir, Glenn David Robert Watson

**Affiliations:** ^1^Department of Neurology, Weill-Cornell Medicine, New York, NY, United States; ^2^Department of Neurology, University of Utah School of Medicine, Salt Lake City, UT, United States; ^3^Department of Neurology, University of Tennessee Health Science Center, Memphis, TN, United States; ^4^LivaNova, Neuromodulation Unit, Houston, TX, United States

**Keywords:** vagus nerve stimulation, drug-resistant epilepsy, medical device, neuromodulation, VNS

## Abstract

The vagus nerve stimulation (VNS) Therapy® System is the first FDA-approved medical device therapy for the treatment of drug-resistant epilepsy. Over the past two decades, the technology has evolved through multiple iterations resulting in software-related updates and implantable lead and generator hardware improvements. Healthcare providers today commonly encounter a range of single- and dual-pin generators (models 100, 101, 102, 102R, 103, 104, 105, 106, 1000) and related programming systems (models 250, 3000), all of which have their own subtle, but practical differences. It can therefore be a daunting task to go through the manuals of these implant models for comparison, some of which are not readily available. In this review, we highlight the technological evolution of the VNS Therapy System with respect to device approval milestones and provide a comparison of conventional open-loop vs. the latest closed-loop generator models. Battery longevity projections and an in-depth examination of stimulation mode interactions are also presented to further differentiate amongst generator models.

## Introduction

The Vagus Nerve Stimulation (VNS) Therapy® System is the first FDA-approved medical device therapy for the adjunctive treatment of drug-resistant epilepsy (DRE) with a proven safety and tolerability profile (1, 2). The system consists of an implantable pulse generator and lead, as well as an external programming system used to change stimulation settings. The pulse generator is a multiprogrammable medical device that delivers electrical signals to the vagus nerve via a lead across various simulation modes. The external programming system allows healthcare providers to change generator settings in addition to visualizing and downloading data collected by the device over time (3). A detailed review of the surgical implant procedure, magnetic resonance imaging safety and compatibility, programming principles, and real-world clinical evidence that supports the therapy's use can be found elsewhere (4–9).

The VNS Therapy's mechanism-of-action involves several pathways (10–19). From a circuit perspective, the anti-convulsive effects of VNS Therapy are thought to be produced by modulating nodes of the “Vagus Afferent Network”: a constellation of brainstem, subcortical, and cortical structures (20). Modulation of this network in epilepsy patients is thought to reduce ictal spread and electrocorticography spatial synchronization (13, 21–23). Neuroimaging methods have further revealed acute and prolonged effects of VNS in thalamic and cortical nodes (15, 24–27). Recent connectomic studies support these observations by demonstrating that the robustness of left-lateralized microstructure and connectivity within limbic, thalamocortical, and hemispheric association fibers reliably predict VNS therapeutic responsiveness (28–31).

The VNS Therapy has evolved through multiple iterations since its inception resulting in software-related upgrades, hardware improvements, and even name: from NeuroCybernetic Prosthesis (NCP) to the VNS Therapy System (3). As of this review, over 125,000 patients have been implanted with the therapy worldwide (6). As new anti-epileptic neuromodulation device technologies enter the therapeutic space, the clinical utility of their programming features and stimulation capabilities must be considered in addition to seizure burden reduction in order to choose the most appropriate therapy for a patient (7–9, 32–37).

Healthcare providers today commonly encounter a range of single- and dual-pin VNS Therapy generators and related programming systems, all of which have their own subtle, but practical differences. In this review, we highlight the developmental genesis of the VNS Therapy's technology in a historical and evolutionary context. Pertinent information from the manufacturer's Physician's Manuals and other resources is consolidated to provide healthcare providers a dedicated reference that compares system components, battery longevity projections, and stimulation modes (3, 5, 38–43).

### Evolution of the VNS Therapy Technology

**Figure 1:**
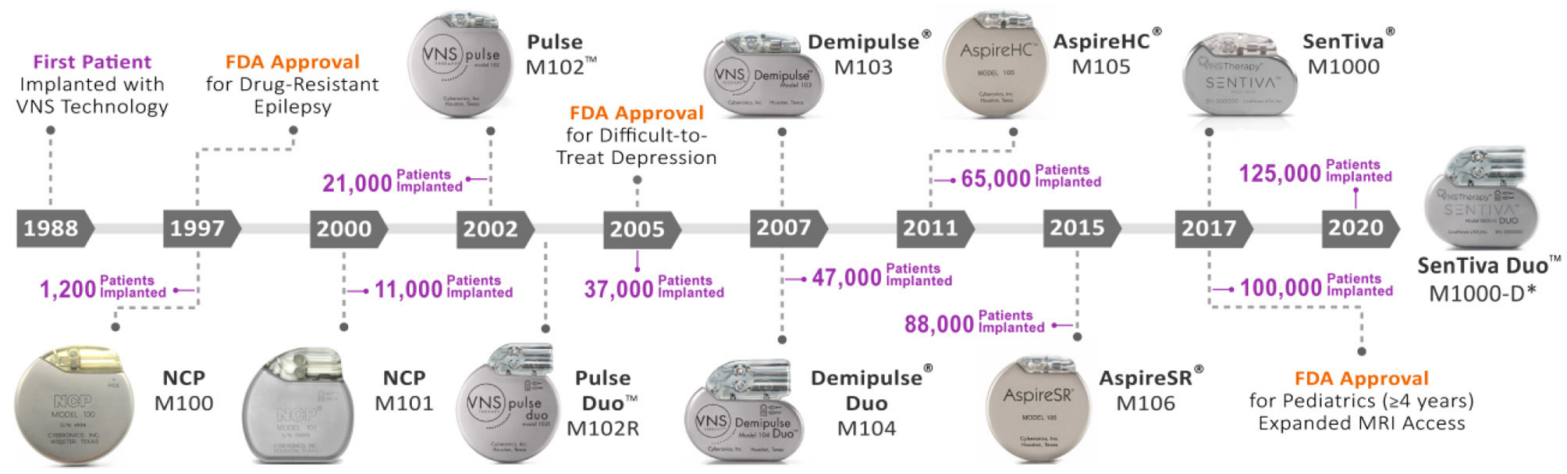
Evolution of the VNS therapy system. Timeline of VNS Therapy System generators with respect to FDA release year and cumulative patient implants worldwide (LivaNova, data on file). In 1988, the first patient was implanted with the VNS Therapy technology by neurologist James Kiffin Penry and neurosurgeon William Bell at the Wake-Forest Bowman Gray School of Medicine in the United States (46). Since then, 10 iterations of the VNS Therapy technology have been released for commercial use. The number of total worldwide patient implants (purple) is shown with respect to generator release year. The VNS Therapy received FDA approval in 1997 for use as an adjunctive therapy in reducing the frequency of partial onset seizures which are refractory to anti-seizure medications in adults and adolescents over 12 years of age. The technology's indication further expanded to include difficult-to-treat depression in 2005 and pediatrics (≥4 years) in 2017 (3). Expanded MRI access up to 3.0 Tesla imaging and allowing use of a transmit body coil for some generators also occurred in 2017 (5). *M1000-D generator is only licensed in Europe as of this review. HC, high capacity; M, model; MRI, magnetic resonance imaging; NCP, NeuroCybernetic Prosthesis; SR, sense and respond.

The widespread use of a peripheral neuromodulation device to treat DRE with the ability to non-invasively adjust parameters was a radical idea at the time of the VNS Therapy's inception. Shortly after the company was founded in 1987, their implantable pulse generator and lead system to stimulate the vagus nerve for DRE received an investigational device exemption for use in clinical studies based on positive experimental results in animals (44, 45). The first vagus nerve stimulator implant in an adult DRE patient was conducted later that year (46). Since then, five generations of the VNS Therapy System technology have been released (Figure 1).

**Figure 2:**
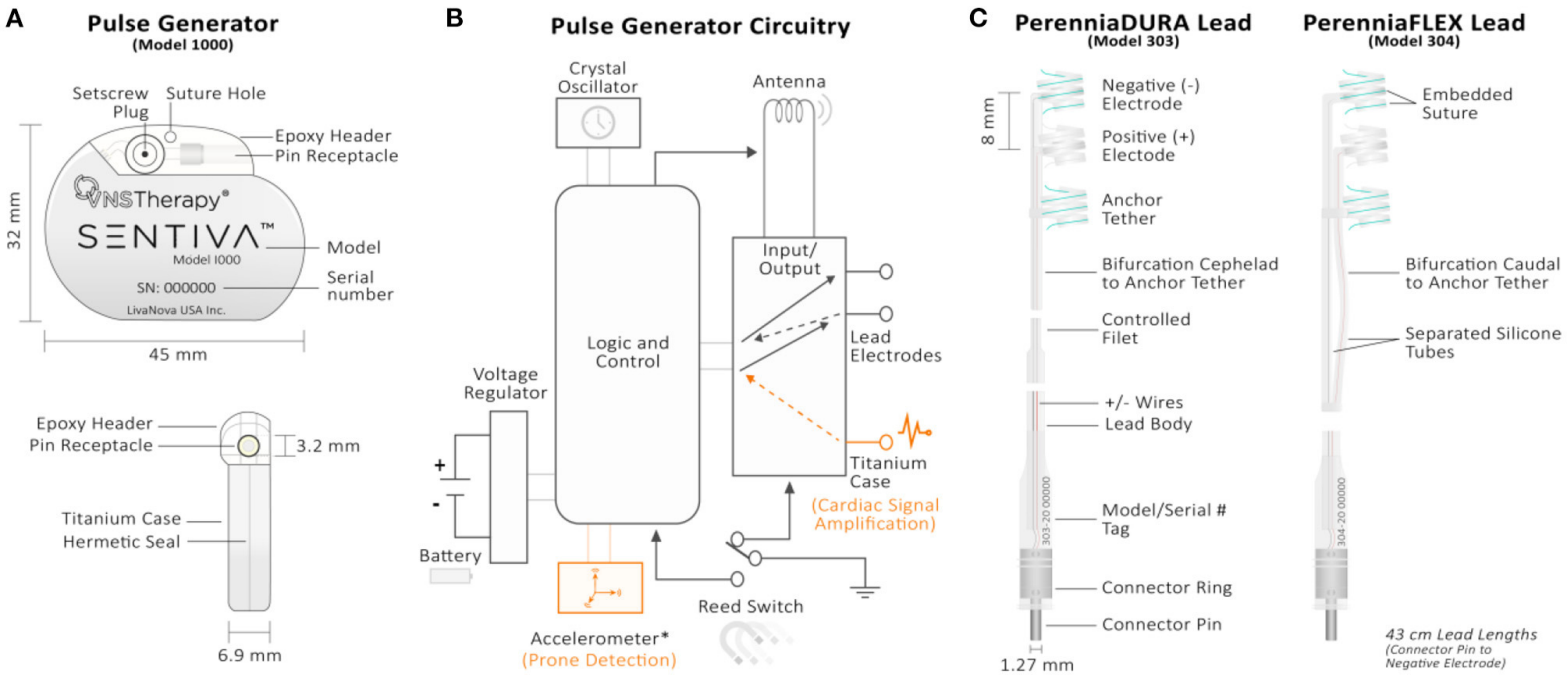
Technical overview of the VNS Therapy System's implantable components. **(A)** Front (top) and side (bottom) views of the M1000 implantable pulse generator. The generator circuitry is hermetically sealed in a titanium case. A hex screwdriver is inserted into the setscrew plug receptacle during surgery to secure the lead's connector pin upon insertion. Model and serial number information are printed on the front face of each generator. **(B)** Pulse generator circuitry schematic. The generator's circuitry includes, (1) crystal oscillator to provide a timing reference; (2) voltage regulator to regulate the system power supply from the battery; (3) antenna to receive programming signals and transmmit telemetry information to the Programming Wand; (4) logic and control that receives and implements programming commands, as well as collects and stores telemetry information (i.e., memory); (5) input/output controller to develop and modulate signals delivered to the lead. It can also allow the traditional VNS to serve as both therapy outputs and sensing inputs; (6) reed switch controlled by swiping the therapy's patient magent. For the M106, M1000, and M1000-D generators, the logic and control also processes sensory information (heart rate) and controls sensory-based therapy outputs (AutoStim). Components unique to the therapy's closed-loop generators (models 106, 1000, and 1000-D) are shown in orange. The input controller of closed-loop generators through the titanium case connection provides cardiac signal amplification. The accelerometer is unique to M1000 and M1000-D generators and provides information related to patient posture for prone event detection. **(C)** Single-pin PerenniaDURA M303 (left) and PerenniaFLEX M304 (right) implantable leads. Each lead features two active helical electrodes (negative and positive) that provides therapy output and a non-active anchor tether electrode used for stabalization purposes. Embedded sutures in the silicone casing of the helical eletrodes allows manipulation with forceps during surgery. The increased flexibility of the M304 lead is provided by the separated silicone tube configuration (right).

**Figure 3:**
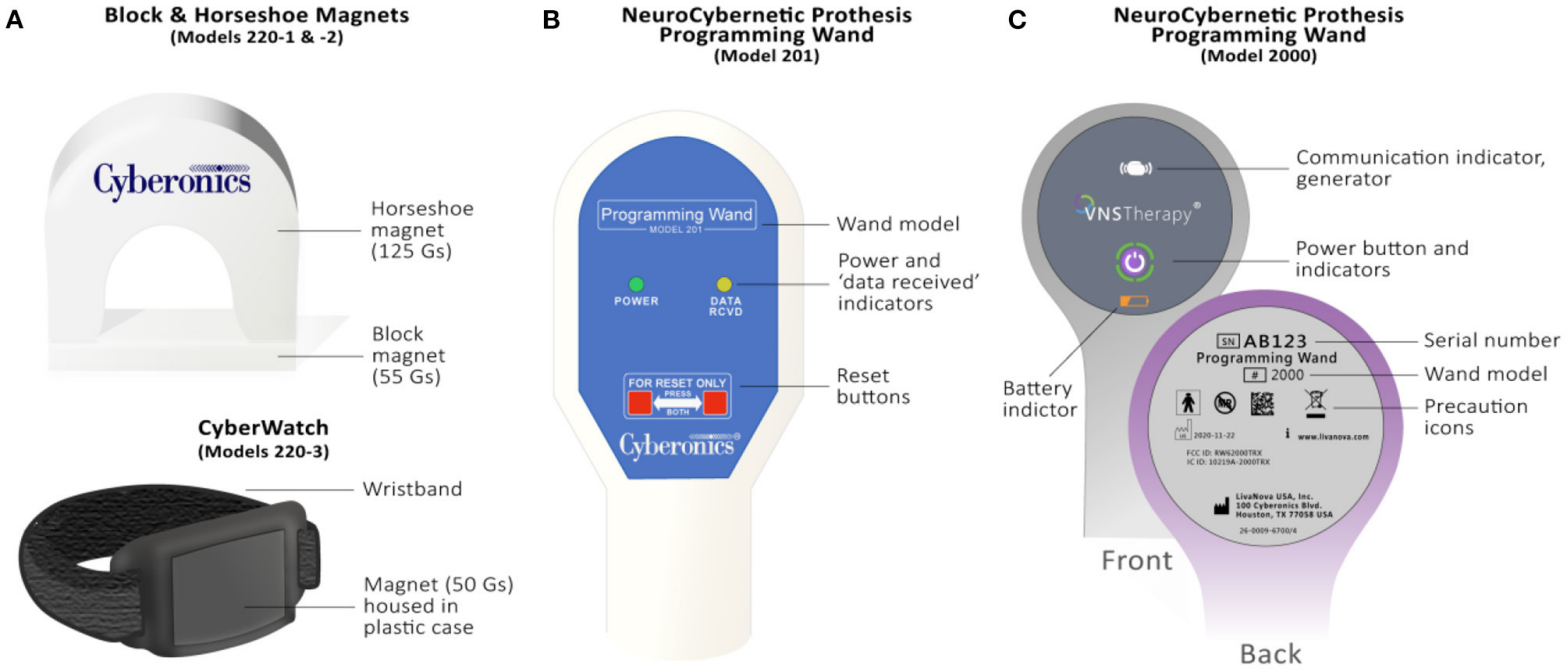
Overview of the VNS Therapy System's patient magnets and Programming Wands. **(A)** Legacy block (Model 220-1) & horseshoe (Model 220-2) magnets (top) and watch-style (Model 220-3) magnet (bottom). Guass (Gs), or magnetic flux density is provided for each magnet. **(B)** M201 Programming Wand. Light indicators on the front signify normal, successful communication (yellow DATA/RCVD light) and a good battery level (green POWER light). Compatible with the M250 “Motion Tablet” Programmer (not shown). The M201 Wand is turned on by pressing two red “RESET” buttons simultaneously. **(C)** Programming Wand M2000. The Wand's power button signifies whether it is powered on (two green lights below power button), connected to the M3000 Programmer (four green lights around power button), or is updating (green lights rotate around power button, v1.1+). Icons on the front of the Wand also signify communication with the generator (white flashing generator icon) and battery status (orange battery indicator if low). The back of the M2000 Wand features a serial number that registers on the M3000 Programmer for identification purposes.

The first NCP generator (M100) was approved by the FDA in 1997 after positive data from two randomized controlled trials (1). The NCP M100 was a dual-pin open loop generator with a wider range of stimulation parameters (programmable up to 143 Hz and 20 mA) and was considered a pioneering device solution for DRE (47). This inaugural generator featured a lithium carbon monofluoride battery housed in a hermetically sealed titanium case (38) (Table 1). Furthermore, the generator featured a reed switch to allow on-demand stimulation after swiping the therapy's block or horseshoe magnets over the generator (Figures 2B, 3A). Apart from generator size, battery life, and software features, most components have been conserved throughout the therapy's evolution (3, 38) (Table 1; Figures 1, 2A,B).

**Table 1:** Feature and specification table for VNS Therapy generator models.

	NCP M100^*^	**NCP M101**	**Pulse M102™**	**Pulse Duo™ M102R**	**Demipulse^®^ M103**	**Demipulse^®^ Duo M104**	**AspireHC^®^ M105**	**AspireSR^®^ M106**	**SenTiva^®^ M1000**	**SenTiva Duo™ M1000-D^†^**
Lead receptacle	Dual-pin	Dual-pin	Single-pin	Dual-pin	Single-pin	Dual-pin	Single-pin	Single-pin	Single-pin	Dual-pin
FDA release year	1997	2000	2002	2003	2007	2007	2011	2015	2017	——–
EU certification mark year	1994	1999	2003	2003	2005	2005	2011	2014	2017	2020
HS-Ti case dimension (mm)	55 ×55 ×13.2	54 ×54 ×10.3	52 ×52 ×6.9	52 ×58.4 ×6.9	45 ×32 ×6.9	45 ×39 ×6.6	52 ×52 ×6.9	52 ×52 ×6.9	45 ×32 ×6.9	45 ×39 ×6.9
Volume (cc)	31	26	14	16	8	10	14	14	8	10
Weight (g)	55	38	25	27	16	17	25	25	16	17
Battery model	9086	9086	2075	2075	2183	2183	2075	2075	2183	2183
Rated capacity (amp-hours)	2.3	2.3	1.7	1.7	1.0	1.0	1.7	1.7	1.0	1.0
X-ray tag code	None	None	CYBX or CYBX-J-XX	CYBX or CYBX-J-XX	CYB A & VNS A	CYB A & VNS A	CYBX	CYBX	LIVN & VNS	LIVN & VNS
Time needed for magnet inhibition (s)	≥ 65	≥ 65	≥ 65	≥ 65	≥ 65	≥ 65	≥ 65	≥ 5	≥ 10	≥ 10

*Feature or specification (left) with respect to VNS Therapy generator model (top). For x-ray tag code CYBX-J-XX, the “XX” signifies the year (e.g., 20 for 2020). Time needed for magnet inhibition is the time the therapy magnet needs to be held over the generator to stop stimulation*.

* *Applies to M100 generators with serial numbers ≥10,000*.

† *M1000-D generator is only licensed in Europe as of this review*.

*HC, high capacity; HS-Ti, hermetically sealed titanium; NCP, NeuroCybernetic Prosthesis; M, model; SR, sense and respond*.

It was not until the Pulse^TM^ M102 that the system changed from a dual-pin to a polarized single-pin system, thereby decreasing the potential of lead communication issues with the generator (39). Further innovation to the generator's design came in 2007 with the Demipulse® M103 and Demipulse Duo® M104: the smallest and lightest VNS Therapy generators (3) (Table 1). Similar to the Pulse^TM^ M102R generator, the M104 generator provided for a smaller dual-pin replacement generator featuring a lead impedance measurement update during diagnostic testing (3). The generator platform went back to a volumetrically larger design in 2011 with the release of the AspireHC® M105 generator that housed a “high capacity” battery (36% longer lifespan compared to the M103 generator) (39).

The release of the AspireSR® M106 “sense and respond” generator in 2015 provided the first responsive, closed-loop form of VNS Therapy. This optional automatic stimulation (AutoStim) Mode detects and responds to a rapid increase in heart rate that may be associated with a seizure (21, 22, 48, 49). The latest SenTiva® M1000 generator preserves the closed-loop AutoStim Mode feature from the M106 generator release but resorted back to the smaller canister specifications of the M103 generator (3) (Table 1). Real-world use of the AutoStim Mode has been shown to increase VNS Therapy therapeutic efficacy in both pediatric and adult patients across varying epilepsy etiologies (50–55). Additionally, patients who elect to switch from a conventional VNS Therapy generator to a closed-loop AutoStim generator (e.g., M106, M1000, M1000-D) can experience additional therapeutic improvement upon generator replacement (50, 52, 56, 57) (Table 2; Figure 1).

**Table 2:** Compatibility of VNS Therapy components.

** 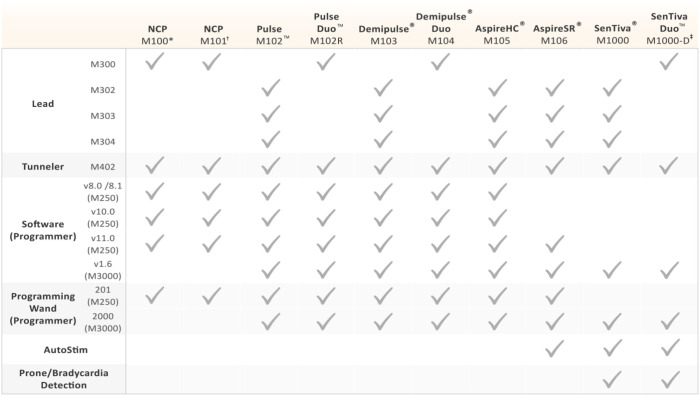 **

*Compatibility of VNS Therapy components (left) with respect to generator model (top). Check mark indicates compatibility*.

* *M100 generators (SN 2,000-9,999) compatible with M250 Programmer software versions 3.8, 4.0, and higher. M100 generators (SN ≥10,000) compatible with M250 Programmer software versions 4.1 and higher*.

† *M101 (all SNs) used M250 Programmer software version 4.4*.

‡ *M1000-D generator is only licensed in Europe as of this review*.

*AutoStim, autostimulation; HC, high capacity; M, model; NCP, NeuroCybernetic Prosthesis; SR, sense and respond*.

The main technological improvements to the M1000 generator are related to communication speed (2,400 baud) and targeted programming options (3). New system features include a wireless programming wand, remote titration capability through Scheduled Programming, Day/Night Programming for patients who, for example, have diurnal fluctuations of seizure activity, and event detections that potentially serve as clinical biomarkers for risk of sudden unexpected death in epilepsy (41, 58–60). A dual-pin version of the M1000 generator named the SenTiva Duo^TM^ M1000-D was recently released and is only licensed within Europe as of this review (Tables 1, 2; Figure 1). This new dual-pin generator model allows patients who have a conventional dual-pin generator model to receive the latest VNS Therapy upon replacement without necessitating a lead revision.

### VNS Therapy Components

#### Implantable Components

##### Pulse Generator

The VNS Therapy generator is a biologically compatible titanium cased device that uses a lithium carbon monofluoride battery (Wilson Greatbatch Ltd) with an open-circuit voltage of 3.3 V and self-discharge rate of <1% per year (3, 38). The generator's header is a Polyurethane (Tecothane^TM^) epoxy that serves as a lead receptacle where the lead pin is inserted (Figures 2A,C).

**Figure 4:**
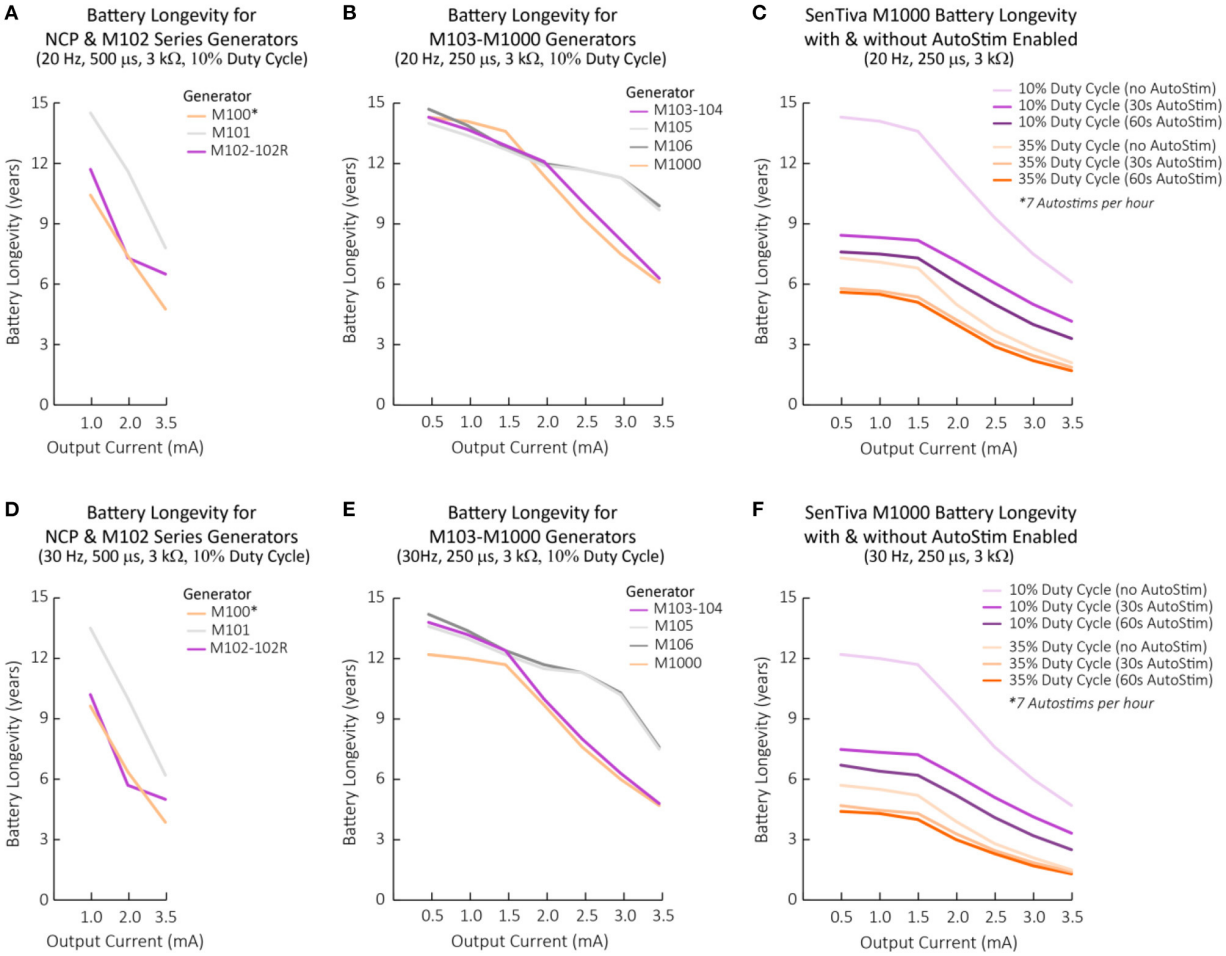
Battery longevity projections for each VNS Therapy generator with respect to programmed stimulation settings. Battery longevity projections under various stimulation parameter settings calculated based on modeling from a generator's beginning of life until EOS (3, 23). **(A–C)** Battery longevity projections for generators programmed at 20 Hz with a typical lead impedance of 3 kΩ across varying output currents. Other stimulation parameters are displayed above the graph in brackets and in the legend, if applicable. **(D–F)** Battery longevity projections for generators programmed at 30 Hz with a typical lead impedance of 3 kΩ across varying output currents. Other stimulation parameters are displayed above the graph in brackets and in the legend, if applicable. **(A,D)** 500 μs was the lowest tested pulse width reported for NCP generators (23). 500 μs was used for M102 series generators for comparison purposes. For the M100 generator, calculations for serial numbers >10,000 were used. **(C,F)** The M1000 battery longevity with and without AutoStim enabled. Calculations based on 7 AutoStims per hour. Note that figure should not be used to exactly predict battery EOS but illustrates the effect of various parameters changes on battery life. AutoStim, autostimulation; EOS, end of service; M, model.

- **Physical Size, Battery Usage, and Identifiers**: The original NCP model generators were the largest, powered by a lithium carbon monofluoride battery with a rated capacity of 2.3 amp-hours (38). The second-largest VNS Therapy generator models (models 102, 102R, 105, 106) have a rated capacity of 1.7 amp-hours, while the smallest models (models 103, 104, 1000, 1000-D) have a rated capacity of 1.0 amp-hours (3). Volumetrically larger generators house larger batteries and, naturally, have longer battery lives dependent upon programmed stimulation settings such as output current and duty cycle (Table 1; Figure 4).
  Besides physical size, some generators can be identified by a model-specific x-ray tag code if interrogation is not possible (i.e., battery death) (3) (Table 1). NCP generator models do not have an x-ray tag and can only be distinguished by either their larger size or orientation of their magnet reed switch as shown on x-ray (diagonal for NCP M100, horizontal for NCP M101 with respect to the header position) (38).
- **Lead Receptacle**: There are two types of lead receptacles based on the number of pins inserted into them: dual-pin (5 mm receptacle inner diameter) and single-pin (3.2 mm receptacle inner diameter) depicted in Figure 2A (3). On dual-pin generator models the bottom, or ventral receptacle labeled (“+”) accepts the positive lead designated with a white marker (38). Each lead is embedded with model and serial number information (Figure 2C).
- **Circuitry**: Each generator model utilizes complementary metal oxide semiconductor (CMOS) integrated circuits, including a microprocessor with varying open-circuit voltages (Table 1). The generator's circuitry is functionally represented in Figure 2B, which includes: (1) crystal oscillator to provide a timing reference; (2) voltage regulator; (3) antenna for telemetry to communicate with the Programming Wand; (4) logic and control that receives and implements programming commands, in addition to collecting and storing telemetry information; (5) input/output controller to develop and modulate signals delivered to the lead; (6) reed switch controlled by swiping the therapy's magnet over the generator (3). Communication speeds have notably improved as the therapy system has evolved: The M1000 generator quadrupuled communication speed compared to previous generators (2,400 vs. 600 baud), improving the transfer of additional information logged by the generator to the programming sofware.
  Some circuitry and processing features are unique to the latest VNS Therapy generators (Figure 2B). For generators that feature AutoStim, the logic and control processes sensory information (heartbeats) and controls cardiac-based therapy outputs (3). The input controller through the titanium case connection also provides amplification of cardiac signals in these generators. The accelerometer is unique to M1000 and M1000-D generators and provides information related to patient posture for prone position event detection.

##### Lead

The lead is the neural interface of the VNS Therapy System. All lead models (300, 302, 303, 304) are available in two sizes (2 and 3 mm inner helical diameter) to ensure optimal electrode fit on different sized nerves (3). The lead has two helical electrodes (anodal and cathodal) and a non-active anchor tether (8 mm separation center to center) for implant stabilization (Figure 2C). Sutures are embedded in the silicone elastomer of each helix to provide surgeons the ability to manipulate the electrodes around the patient's nerve with Cushing forceps (4) (Figure 2C).

The original design of the lead was developed with strands of 1-mm stainless-steel wire (61). Shortly thereafter, the lead wire was changed to a trifilar cobalt-chromium-nickel alloy (MP35n) in response to a 90% incidence of breakage (47). The MP35n is a carbonless alloy with exceptional corrosion- and fatigue-resistant properties found to last 170x's longer than the original stranded stainless-steel wire.

Both NCP generators were compatible with the original, but now extinct dual-pin M300 lead: a 43 cm long biocompatible platinum wire with an inner helix diameter of 2 mm and individual helix lengths of 7 mm (61). Currently available single-pin leads (302–304) are fashioned from the dual-pin M300 lead: a platinum-iridium wire with silicone insulation (39). It is important to note that the first single-pin lead in the series (M302) is only available outside of the United States.

- **Lead Connector Pin(s)**: Two types of lead pin configurations can be inserted into the header's receptacle: a dual-pin lead configuration (model 300) for generators with dual-pin receptacles/headers (models 100, 101, 102R, 104, 1000-D) and a single-pin lead configuration (models 302, 303, 304) for generators with single-pin receptacle/headers (models 102, 103, 105, 106, 1000). The connector is a 300 series stainless-steel pin (1.27 mm diameter) with a rated connector retention strength of > 10 N. The lead body (2 mm diameter, 43 cm length) contains a silicone insulation with either a trifilar (model 303) or quadfilar (models 302, 304) MP35n alloy conductor construction (39).
- **Lead Resistance**: Lead resistance is measured from pin to electrodes: 120–180 Ω for models 300, 302, 304, and 180–250 Ω for the model 303 (39). When testing lead impedance with the Programmer, an acceptable lead impedance range is from 600 to 5,300 Ω. Impedance values outside of this range can signify a short-circuit, lead break, or improper insertion of the connector pin, among other reasons (3). Because NCP and M102 series generators are not capable of measuring lead impedance, a DC-DC converter code is reported indicative of the estimated lead impedance at 1 mA output current and 500 μs pulse width (e.g., DC-DC code of “7” signifies high impedance) (38).
- **Lead Durability**: The 5- and 10-year survival of the M300 dual-pin lead is 94.4 and 86.4%, respectively (3). The single-pin M302 lead has a similar 5-year (93.1%) and 10-year (86.5%) survival to its dual-pin predecessor. The company subsequently manufactured the M303 lead to address long-term durability issues with the M302 lead. Aptly named the PerenniaDURA, the M303 lead is 17 x's stronger than the M302 lead with a reported 5-year survival of 97.6% (Figure 2C). The M304 lead (PerenniaFLEX) was released to address the M303's stiffness and provides more flexibility during surgical implantation because of its separated silicone tube design (Figure 2C). The M304 lead is 3.5 x's more durable than the M302 lead and was found to have a similar 5-year survival rate (97.1%).

#### Non-implantable Components for Surgeons

- **Tunneler** (Model 402): The tunneler is a single use surgical instrument for tunneling the lead subcutaneously between the neck incision site and the chest pocket made before generator insertion (42). The tunneler has a stainless-steel shaft (34 cm) and a bullet tip (7.9 mm) to aid tunneling. The tunneler packet comes with two fluorocarbon polymer sleeves to tunnel either a single-pin (inner/outer diameter 3.4/74.7 mm; length 26.5 cm) or dual-pin (inner/outer diameter 6.4/7.9 mm; length 28 cm) lead. Surgeons can manually bend the tunneler up to 25° to aid lead tunneling.
- **Accessory Pack** (Model 502): The Accessory Pack contains replacement components for the generator and lead that may become unusable during surgery (43). The Accessory Pack includes test resistors, a hex screwdriver, and four radiopaque silicon tie-downs. The resistor is inserted into the generator (single- or dual-pin) to mimic lead impedance for intraoperative testing of generator functionality. The hex screwdriver is used to loosen, retract, and tighten the setscrew to allow the escape of backpressure created by inserting the lead connector pin into the receptacle of the generator. Four tie downs also come in the Accessory Pack made from radiopaque silicone (5.7 × 7.7 mm) provided to secure excess lead and help form the strain-relief bend and loop that provides the slack necessary for neck movement.

#### Non-implantable Components for Patients and Healthcare Providers

##### Patient Essentials Kit With Therapy Magnets

(Model 220): The Patient Essentials Kit is given to patients post-surgery and includes a patient manual, two patient identification cards, and two therapy magnets with a wristband (watch-style) and belt clip (pager-style) (3). The VNS Therapy magnet is used for one-way communication with the generator to provide on-demand stimulation. Patients can use the magnet in several situations, such as helping to abort or lessen the intensity of an oncoming seizure, temporarily inhibiting stimulation, testing generator function, and habituating to newly programmed stimulation settings (3). The magnet can also be used to reset the generator in combination with the Programming Wand. The therapy's magnet has had two iterations since the technology's inception:

- **Block** (model 220-1) and **Horseshoe** (model 220-2) **Magnets**: Released during the NCP generator era, the horseshoe magnet (2 × 2 × 0.8 inches, alnico 5 core material) and block magnet (2 × 1 × 0.675 inches, strontium ferrite core material) produced 125 and 55 gauss minimum magnetic flux density, respectively (Figure 3A) (LivaNova, internal communication).
- **CyberMagnet** (Model 220-3 and 220-4): The CyberMagnet was introduced in 2001 to replace the block and horseshoe magnet designs (3). This magnet is made from Neodymium grade 35 (NdFeB-35) encased in a polypropylene copolymer and produces 50 gauss minimum magnetic flux density at 1 inch from its surface (Figure 3A). Magnets provided in the Patient Essentials Kit come with a wristband (“CyberWatch,” model 220-3) and a clip to attach to a belt or belt loop (“CyberPager,” model 220-4) with a quick release mechanism. These updates provide effortless accessibility and decrease the likelihood of patients losing their magnet.
  Temporary suspension of the therapy can be achieved by holding the magnet over the generator. To stop stimulation long-term, the patient can leave the magnet over the generator by taping the magnet to their chest by using an elastic, wraparound bandage. For generator models 102R, 103, 104, and 105, >65 s is needed to suspend therapy with the magnet placed over the generator, whereas less time is needed for the M106 (>3 s) and the M1000 (>10 s) generators (3, 39). Stimulation restarts when the magnet is removed from the generator.

##### Programming Wand

The Programming Wand is a handheld device that transmits information between the generator and Programmer via telemetry (41) (Figures 2B, 3B,C). Successful programming and communication are most likely if the surface of the Programming Wand's head is within one inch of either of the generator's flat surfaces. The Wand is also used with the therapy magnet to reset the generator.

- **NCP Programming Wand** (Model 201): The inaugural M201 NCP Programming Wand (9.76 in. length, 560 g, ABS plastic) was powered by a 9V battery and communicated with the Programmer through a cable (10 ft., RS-232 serial) connected to a standard DB9 (9-pin) plug (40) (Figure 3B). The M201 Programming Wand has an internal oscillator that runs at a frequency of 97 kHz (+/– 10 kHz) when it is active and is compatible with all generators except M1000 and M1000-D generators (Table 2). The Wand can receive a nominal 40 kHz magnetically coupled signal from the generator (−6.92 effective radiated power at 3 meters) and light indicators on the front signify normal, successful communication (yellow DATA/RCVD light) and a good battery level (green POWER light). Briefly, the M201 Wand is turned on by pressing two red “RESET” buttons simultaneously (Figure 3B). Electromagnetic interference upon powering is signified by both yellow DATA/RCVD and green POWER lights coming on. To reset the generator with the M201 Programming Wand, one must continuously press down on the two red POWER buttons for at least 30 s while holding the magnet over the generator.
- **Model 2000 Programming Wand:** Released with the M1000 generator in 2017, the M2000 Programming Wand is compatible with all generators except the NCP series (41) (Table 2). The Wand takes two AA lithium or alkaline batteries and can wirelessly communicate with the M3000 Programmer up to three meters (Bluetooth 2.1: 10.4 dBm transmitter power, 2402–2480 MHz operation frequency and receiver bandwidth). A USB Type-C backup cable (2.87 m) is provided if communication errors between the Wand and Programmer are experienced. The companion M3000 Programmer will display the Wand's serial number when it is within range. The Wand's power button signifies whether it is powered on (two green lights below power button), connected to the Programmer (four green lights around power button), or is updating (green lights rotate around power button, v1.1+) (Figure 3C). Icons on the front of the Wand also signify communication with the generator (white flashing generator icon) and battery status (orange battery indicator if low). Once powered on, the Wand will automatically power down after 2 min of inactivity to conserve battery.

##### Programmer and Software

The Programmer is a hand-held device or tablet-style computer with a touch-screen interface that connects to the Programming Wand (via a cable or wirelessly if applicable) and runs the programming software. The software allows healthcare providers to interrogate, overview, and/or change the VNS Therapy output parameters, as well as assess lead function and export session reports (Table 2).

**Figure 5:**
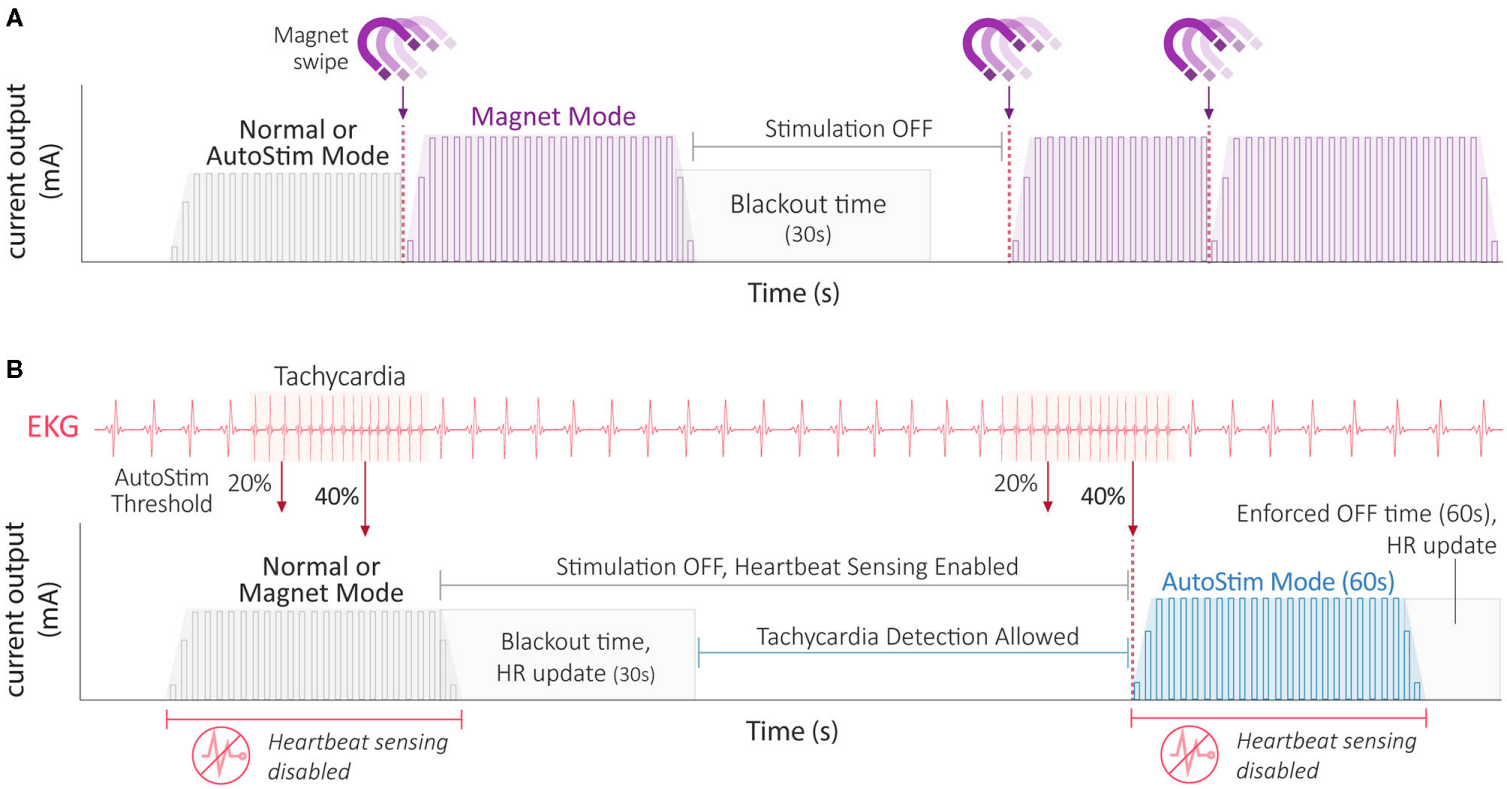
VNS Therapy stimulation mode interactions. **(A)** Interaction amongst stimulation modes during a therapy magnet swipe (purple icon). A magnet swipe interrupts both Normal Mode and AutoStim Mode stimulation (left). A magnet swipe during a Magnet Mode stimulation effectively resets the Magnet Mode stimulation period (right). Note the stimulation blackout time of 30 s to prevent overstimulation. **(B)** Interaction of stimulation modes with respect to tachycardia-triggered AutoStim Mode. Detected tachycardic events at varying AutoStim thresholds are shown superimposed upon an EKG trace. Because heartbeat sensing is disabled during a stimulation event, AutoStim Mode stimulation is not possible despite the heart rate surpassing the set AutoStim threshold (e.g., 40%). The Tachycardia Detection Algorithm is updated with heart rate information for 30s after stimulation. Also note the stimulation blackout time of 30s to prevent overstimulation where AutoStims cannot occur. Tachycardia detection, and therefore AutoStim is allowed after the blackout period. When a patient's relative heart rate surpasses the set AutoStim threshold (e.g., 40%) during an allowable time, AutoStim is triggered (right). Following an AutoStim event, an ‘enforced OFF time' equal to the length of the AutoStim event ensures a ≤ 50% duty cycle. Note that AutoStim Mode is only available on M106, M1000 and M1000-D generators. AutoStim, autostimulation; EKG, electrocardiogram; HR, heart rate.

- **Model 250 Programmer:** Known as the **“**Motion Tablet,” this Dell Programmer ran in a Windows environment and came with both an SD card (4 GB) to download patient information and a stylus for healthcare providers to interface with the Programmer (40). The M250 Programmer played rapid, musical tones to signify successful or unsuccessful completion of an interrogation or programming operation. A switch was later made to a solid-state hard drive to eliminate the need to reconfigure setup upon loss of power, and touchscreen capabilities with or without the use of a stylus.
- **Model 3000 Programmer:** The M3000 Programmer is an Hewlard Packard touch-screen tablet compatible with the M2000 Programming Wand (41) (Table 2). Much like the M250 Programmer, successful interrogation, diagnostics, or applied changes are indicated by the sound of musical notes. A 32 GB micro-USB storage drive is provided in the Programmer's hand strap to download patient information and to allow sharing of custom protocols amongst healthcare providers. Guided Programming on the M3000 Programmer also allows therapy titration using an FDA-approved protocol (3).
- **Battery Status Indicators:** For NCP and M102 series generators, the programming software has one battery status indicator: Near End of Service (N EOS) (38, 39). For all other generator models, the remaining battery power is indicated by a battery icon on the Programmer's home screen that represents the generator's battery voltage level (3, 39). No warning message is displayed for battery power remaining >18% for M103/104 generators and >11% for M105-1000 generators. Three unique battery life indicators will be displayed thereafter: Intensified Follow-up Indicator (IFI; 8–18% for M103/104 generators, 5–11% for M105-1000 generators), N EOS (0–8% for M103/104 generators, 0–5% for M105-1000 generators), and EOS (0%). At the level of N EOS, it is recommended that the pulse generator be replaced as soon as possible. If the pulse generator is not replaced in a timely manner, it will eventually lose the ability to communicate with the programming software and poses the risk of reducing any therapeutic effect gained with the therapy (62). Lastly, for NCP and M102 series generators, a consideration should be made not to use frequencies of 5 Hz or less for long-term stimulation because these low frequencies have been shown to generate an electromagnetic trigger signal that results in excessive battery depletion.
- **Heartbeat Detection and AutoStim Threshold (M106, M1000, and M1000-D generators):** The M106, M1000, and M1000-D generators provide an optional AutoStim Mode feature that works in conjunction with Normal Mode stimulation (Figure 5). AutoStim is a responsive, closed-loop form of stimulation activated by a rapid increase in heart rate (tachycardia) (63). To provide this form of stimulation, the VNS Therapy System accurately detects a patient's heartbeat measured from the lead electrode to the generator: an electrocardiogram (EKG) vector unique to the VNS Therapy and used as the input for its tachycardia detection algorithm (64). The algorithm's goal is to reliably detect the R-wave of a patient's heartbeat with a minimum amplitude of 0.4 mV processed by the generator's logic and control (Figure 2B). Healthcare providers can change the sensitivity for Heartbeat Detection from 1 to 5 (with “1” being the least sensitive and “5” being the most sensitive) within the programming software (41). Implant locations outside of the left subclavicular region and above rib 4, and/or inadequate heartbeat detection configuration could negatively impact R-wave detection performance results. In general, a larger distance between the generator and lead electrode, or positioning the generator closer to the heart will result in a better signal (64).
  Healthcare providers can further customize the AutoStim Mode to meet patients' needs by adjusting its threshold sensitivity. Available from 20% (most sensitive) to 70% (least sensitive), this floating detection threshold automatically adjusts to the patients' underlying heart rate activity (41). Briefly, the floating detection threshold is the ratio of the background and foreground heart rates calculated by taking the moving average of instantaneous heart rate samples within a 5 min and 10 s window, respectively. The AutoStim Mode is enabled if this relative heart rate change exceeds the set threshold percentage above the patient's background heart rate.

## Conclusions

Provided herein is a practical reference for healthcare providers that compares VNS Therapy hardware and software components in a technological context. The VNS Therapy System technology has undergone numerous hardware upgrades to reduce the implantable pulse generator's size and weight, increase implantable lead durability, and optimize the generator's circuitry to detect and process cardiac signals. Likewise, software improvements include increased communication speeds, automatic scheduled stimulation dose changes, the ability to perform cardiac-triggered stimulation, and the incorporation of event detection markers to inform clinical decision making. Battery longevity is reliant on both programmed stimulation features and whether the optional AutoStim Mode feature is enabled. Overall, this review serves as a valuable resource as this anti-epileptic neuromodulation technology continues to evolve.

## Author Contributions

PA participated in the concept in the review. All authors constructed figures and tables and wrote the first and final drafts of the manuscript.

## Conflict of Interest

GW is an employee of LivaNova. PA and SA have participated in clinical trials for LivaNova. The remaining author declares that the research was conducted in the absence of any commercial or financial relationships that could be construed as a potential conflict of interest.

## Publisher's Note

All claims expressed in this article are solely those of the authors and do not necessarily represent those of their affiliated organizations, or those of the publisher, the editors and the reviewers. Any product that may be evaluated in this article, or claim that may be made by its manufacturer, is not guaranteed or endorsed by the publisher.
